# New chronic disease medication prescribing by nurse practitioners, physician assistants, and primary care physicians: a cohort study

**DOI:** 10.1186/s12913-016-1569-1

**Published:** 2016-07-27

**Authors:** Zachary A. Marcum, Johanna E. Bellon, Jie Li, Walid F. Gellad, Julie M. Donohue

**Affiliations:** 1 Department of Pharmacy, School of Pharmacy, University of Washington, 1959 NE Pacific St, Box 357630, Seattle, WA 98102 USA; 2 Wolff Center at UPMC, Ross Building, Room 219, 4601 Baum Blvd, Pittsburgh, PA 15213 USA; 3 Department of Health Policy & Management, Graduate School of Public Health, University of Pittsburgh, 130 De Soto Street, Pittsburgh, PA 15261 USA; 4 VA Pittsburgh Healthcare System and Department of General Internal Medicine, School of Medicine, University of Pittsburgh, 200 Meyran Avenue, Parkvale Building, Pittsburgh, PA 15213 USA

**Keywords:** Health services research, Pharmacoepidemiology, Primary care, Work force

## Abstract

**Background:**

Medications to treat and prevent chronic disease have substantially reduced morbidity and mortality; however, their diffusion has been uneven. Little is known about prescribing of chronic disease medications by nurse practitioners (NPs) and physician assistants (PAs), despite their increasingly important role as primary care providers. Thus, we sought to conduct an exploratory analysis to examine prescribing of new chronic disease medications by NPs and PAs compared to primary care physicians (PCPs).

**Methods:**

We obtained prescribing data from IMS Health’s Xponent™ on all NPs, PAs, and PCPs in Pennsylvania regularly prescribing anticoagulants, antihypertensives, oral hypoglycemics, and/or HMG-Co-A reductase inhibitors pre- and post-introduction of five new drugs in these classes that varied in novelty (i.e., dabigatran, aliskiren, sitagliptin or saxagliptin, and pitavastatin). We constructed three measures of prescriber adoption during the 15-month post-FDA approval period: 1) any prescription of the medication, 2) proportion of prescriptions in the class for the medication, and 3) time to adoption (first prescription) of the medication.

**Results:**

From 2007 to 2011, the proportion of antihypertensive prescriptions prescribed by NPs and PAs approximately doubled from 2.0 to 4.2 % and 2.2 to 4.9 %, respectively. Similar trends were found for anticoagulants, oral hypoglycemics, and HMG-Co-A reductase inhibitors. By 2011, more PCPs had prescribed each of the newly approved medications than NPs and PAs (e.g., 44.3 % vs. 18.5 % vs. 20 % for dabigatran among PCPs, NPs, and PAs). Across all medication classes, the newly approved drugs accounted for a larger share of prescriptions in the class for PCPs followed by PAs, followed by NPs (e.g., dabigatran: 4.9 % vs. 3.2 % vs. 2.8 %, respectively). Mean time-to-adoption for the newly approved medications was shorter for PCPs compared to NPs and PAs (e.g., dabigatran, 7.3 vs. 8.2 vs. 8.5 months; *P* all medications <0.001).

**Conclusions:**

PCPs were more likely to prescribe each of the newly approved medications per each measure of drug adoption, regardless of drug novelty. Differences in the rate and speed of drug adoption between PCPs, NPs, and PAs may have important implications for care and overall costs at the population level as NPs and PAs continue taking on a larger role in prescribing.

**Electronic supplementary material:**

The online version of this article (doi:10.1186/s12913-016-1569-1) contains supplementary material, which is available to authorized users.

## Background

Since the 1977 passage of the Rural Health Clinic Services Act, nurse practitioners (NPs) and physician assistants (PAs) have been authorized by Medicare and Medicaid to be reimbursed as providers [1]. Although all states allow NPs and PAs to practice in some capacity, scope-of-practice laws vary widely by state [2, 3]. Nationally, approximately 56 % of physician group practices and 40 % of independent providers utilize NPs and PAs in their practices, with continued growth expected due to a confluence of factors – fewer medical students choosing primary care, increasing health care demand from a growing and aging population, and a broad increase in access to care through the Affordable Care Act [1, 4, 5]. NPs and PAs generally perform 80 % of the functions of physicians, and studies to-date indicate that their quality measures and patient satisfaction are equal or greater than physicians’ for care within their scope-of-practice [1, 6–8].

One of the most important roles of a primary care provider – NP, PA, or primary care physician (PCP) – is to manage chronic diseases, and pharmacotherapy is the mainstay of treatment. National estimates for the US indicate that approximately 70 % of primary care office visits result in a medication being prescribed [9]. A review of state NP and PA regulations from 2001 to 2010 found that most states have loosened regulations over time, granting greater autonomy to NPs and PAs, particularly with respect to prescriptive authority [10].

Some of the most widely prescribed chronic medication classes are those to treat hyperlipidemia and prevent the development and progression of ischemic heart disease, stroke, diabetes, and heart failure, which have greatly reduced cardiovascular disease-related morbidity and mortality [11, 12]. However, diffusion of these therapies across the US is sub-optimal. There is both ‘under-diffusion’ of evidence-based therapies and ‘over-diffusion’ of new drugs that are more costly but no more effective than existing therapies. Diffusion is known to vary by physician specialty, with PCPs typically adopting new drugs more slowly than physicians with sub-specialty training [13]. However, it is not known how NPs and PAs utilize newly-approved drugs compared to PCPs despite their increasingly important role as primary care providers. Differential uptake of new chronic disease medications by NPs and PAs compared to physicians in primary care has potential implications for both cost and quality of care. Thus, we sought to conduct an exploratory analysis to examine the prescribing of new cardiovascular and diabetes medications by NPs and PAs relative to that of PCPs.

## Methods

### Data sources and sample

We used IMS Health’s Xponent™ prescription database to characterize NP, PA, and PCP prescribing of five new chronic disease medications (representing four medication classes), and IMS Healthcare Organization Services™ (HCOS) database for provider specialty and organizational affiliations. While data exist for physician characteristics, such as age, medical school, and residency program, in the American Medical Association Physician Masterfile, an equivalent dataset does not exist for NPs and PAs. As a result, we were unable to measure these provider-level characteristics in our sample. Xponent™ directly captures 86 % of all US prescriptions filled in retail pharmacies and utilizes a patented proprietary projection method to represent 100 % of prescriptions filled in these outlets [14]. We obtained monthly provider-level data on all prescriptions of interest dispensed in Pennsylvania between January 1, 2007 and December 31, 2011. Of note, certified registered NPs and PAs in Pennsylvania are both authorized to prescribe medical and therapeutic treatments. Xponent™ contains limited patient-level information, including the source of payment (Medicare, Medicaid fee-for-service, commercial insurance, cash or uninsured) and patient age, with no individual patient identifiers. We obtained information on provider specialty, provider sex, and organizational affiliations (e.g., medical group) from IMS Health’s HCOS database. HCOS data were used to identify primary care medical groups and to specify the number of providers within each medical group.

### Study cohort

We compiled data for four cohorts of prescribers – one for each of the medication classes under investigation – including prescribers of oral anticoagulants, antihypertensives targeting the renin-angiotensin-aldosterone system, oral hypoglycemics, and HMG-CoA reductase inhibitors among the three prescriber types (i.e., NPs, PAs, and PCPs). We excluded those who never prescribed a medication from the class of interest during the study period. Because we were interested in primary care prescribing behavior, we excluded those providers not practicing in a primary care practice as well as those with missing values for National Provider Identifier or sex.

### Study medications

We measured new medication adoption in our sample for each of five new medications in the four classes of interest (dabigatran, FDA approval: 10/2010; aliskiren, approved 3/2007; sitagliptin and saxagliptin, approved 10/2006 and 7/2009, respectively; and pitavastatin approved 8/2009). These drugs were of varying novelty – as assessed by their benefit/risk profiles and mechanisms of action – and differed in their order of entry in their respective therapeutic classes. For example, dabigatran was the first of a new class of oral anticoagulants with improved efficacy and potentially lower risk of bleeding compared to warfarin in patients with atrial fibrillation [15, 16]. Sitagliptin and saxagliptin were part of a new class of oral hypoglycemic agents that target a different physiologic pathway than other diabetes medications and were purported to have minimal risk of hypoglycemia and weight gain compared to sulfonylureas with comparable efficacy [17]. Aliskiren was the first in a new class of agents for hypertension, joining two others (i.e., angiotensin-converting enzyme inhibitors and angiotensin II receptor blockers) that inhibit the renin-angiotensin-aldosterone system; comparative efficacy and safety among the antihypertensive classes is largely unknown. Pitavastatin was the seventh ‘statin’ approved in the US and represents the most minor therapeutic advance among the study drugs [18]. This variation in medication classes and novelty allowed us to assess whether any differences between NP, PA, and PCP prescribing were specific to a medication class or consistent across multiple classes.

### Outcome measures

The decision to adopt a new drug is multifaceted. A prescriber first needs to learn about a new drug and then decide whether to use it and how frequently to prescribe it. Therefore, we constructed three measures: 1) any prescription of the newly approved medication in the final year of the study period (2011), 2) proportion of new medication among the medication class, and 3) time to first adoption of the newly approved medication. Time to first adoption – defined as the time from FDA approval to first prescription of the new drug – provides a measure of speed of adoption, while the other two measures identify the extent of provider adoption over time [19]. In order to assess NP and PA prescribing trends in general, we also measured the share of prescribing within the drug classes of interest accounted for by NPs and PAs compared to PCPs over time.

### Statistical analysis

Our analysis followed three steps. First, for each of the four chronic disease medication classes, we described characteristics of NPs, PAs, and PCPs who were regular prescribers of the medication class as a frequency (percentage) for each variable. We used Chi-square tests to assess differences in characteristics across provider types, and Wilcoxon rank sum tests for non-normally distributed variables. Second, for each of the four chronic disease medication classes, we measured the proportion of all medications within the class prescribed by the three provider types (i.e., NPs, PAs, and PCPs) across all years. Third, we estimated new drug adoption among the three provider types by assessing the three measures of adoption previously described. To assess time to first adoption, we used the Kaplan-Meier method to compute the proportion of providers who had adopted the new drug in the 15 months post-FDA approval. The date of first prescription of each newly approved medication in the dataset was used as the index date.

Given evidence suggesting the potential for sex differences in new drug adoption by providers [20], we conducted post-hoc sensitivity analyses. Because sex was almost perfectly correlated with provider type, we could not control for it in multivariable analysis. Thus, we repeated all primary analyses described above on only female providers across the three provider types. We used SAS version 9.3 (SAS Institute, Cary, NC, USA) for analyses and the Stata version 11.0 (StataCorp, College Station, TX, USA) for the Kaplan-Meier graphs.

## Results

### Characteristics of the study sample

We identified more than 5000 NPs, PAs, and PCPs in Pennsylvania who prescribed each chronic disease medication category in 2007–2011 (oral anticoagulants, *n* = 5299; select antihypertensives, *n* = 5514; oral hypoglycemics, *n* = 5510; HMG-CoA reductase inhibitors, *n* = 5454; and all four classes, *n* = 5177). Among the medication classes with the most prescribers – antihypertensives – NPs and PAs were more likely than PCPs to be female (93 % of NPs and 77 % of PAs vs. 34 % of PCPs, *P* < 0.001) (Table 1). NPs and PAs were also more than twice as likely to practice in a rural setting (21 % of NPs and 27 % of PAs vs. 12 % of PCPs, *P* < 0.001). PCPs were slightly more likely to prescribe to older patients (with age ≥65 years representing 52 % of PCP patients vs. 41 % of NP patients and 40 % of PAs, *P* < 0.001). PCPs prescribed a larger annual volume of antihypertensive prescriptions compared to NPs and PAs (2011 mean: PCPs, 1127.2; NPs, 433.3; PAs, 423.8; *P* < 0.001). Similar results were found for the other three medication classes (see Additional file 1: Tables S1-S3).

**Table 1:** Characteristics of nurse practitioners, physician assistants, and primary care physicians who prescribed select antihypertensive^a^ prescription medications in Pennsylvania, 2011^b^

Characteristics	Nurse practitioner	Physician assistant	Primary care physician
	*N* = 504	*N* = 591	*N* = 4419
Provider sex, %	--	--	--
Female	93.1	76.8	34.2
Provider setting, %	--	--	--
Rural	20.8	27.4	11.9
Provider prescribing	--	--	--
Annual prescription volume, mean (sd)	433.3 (496.6)	423.8 (496.0)	1127.2 (853.8)
Patient age, %	--	--	--
<64	59.1	60.0	47.7
65–74	19.9	20.2	22.6
75–84	13.4	13.0	18.5
85+	7.7	6.8	11.2
Source of payment for prescription, %	--	--	--
Cash	5.3	5.5	5.3
Commercial	55.2	54.8	54.9
Medicaid	8.7	9.1	5.4
Medicare	30.7	30.6	34.3
Primary care medical group	--	--	--
Number of practice sites	550	569	3277
Number of providers per site, mean (sd)	45.1 (160.1)	47.2 (148.9)	20.7 (89.0)

^a^Antihypertensive medications included those targeting the renin-angiotensin-aldosterone system, including angiotensin-converting enzyme inhibitors, angiotensin II receptor blockers, and direct renin inhibitors; ^b^ *P* <0.001 for comparisons across providers for all variables

### Prescribing patterns over time

From 2007 to 2011, the proportion of all prescriptions in these classes written by NPs and PAs increased substantially. Among select antihypertensives, the proportion of prescriptions written by NPs and PAs approximately doubled from 2.0 to 4.2 % and 2.2 to 4.9 % among NPs and PAs, respectively (Table 2). Conversely, the proportion of all antihypertensive prescriptions accounted for by PCPs decreased from 95.9 to 91.0 % (Table 2). Similar results were found for the other three medication classes.

**Table 2:** Proportion of cardiovascular prescriptions ordered by nurse practitioners, physician assistants, and primary care physicians over time, 2007–2011

	2007	2008	2009	2010	2011
Antihypertensives					
Total prescriptions, *N*	2,116,048	2,182,069	2,188,136	2,248,183	2,272,489
NP (%)	2.0	2.4	3.0	3.8	4.2
PA (%)	2.2	2.8	3.4	4.2	4.9
PCP (%)	95.9	94.8	93.6	92.1	91.0
Anticoagulants					
Total, *N*	261,698	264,176	267,630	278,745	291,524
NP	1.5	1.9	2.3	3.2	3.4
PA	1.4	2.1	2.8	3.6	4.1
PCP	97.1	96.0	94.9	92.6	92.6
Oral hypoglycemics					
Total, *N*	1,292,094	1,293,618	1,302,716	1,315,114	1,301,535
NP	1.9	2.3	2.9	3.7	4.0
PA	2.1	2.7	3.3	4.1	4.8
PCP	96.0	95.0	93.8	92.3	91.2
HMG-CoA Reductase inhibitors					
Total, *N*	1,294,269	1,319,042	1,359,293	1,411,180	1,455,255
NP	2.1	2.6	3.3	4.1	4.4
PA	2.3	2.8	3.5	4.4	5.1
PCP	95.6	94.5	93.2	91.6	90.5

*Abbreviations*: *NP* nurse practitioner, *PA* physician assistant, *PCP* primary care physician

### New drug adoption

**Fig. 1:**
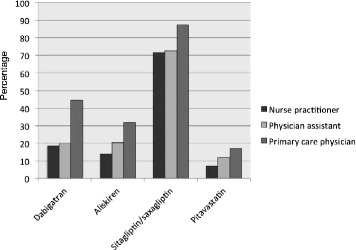
Percent of each provider type with any prescription for a newly approved medication in 2011^a^. ^a^Prevalence of any prescription of newly approved chronic disease medications among primary care providers prescribing any drug from the medication class

**Fig. 2:**
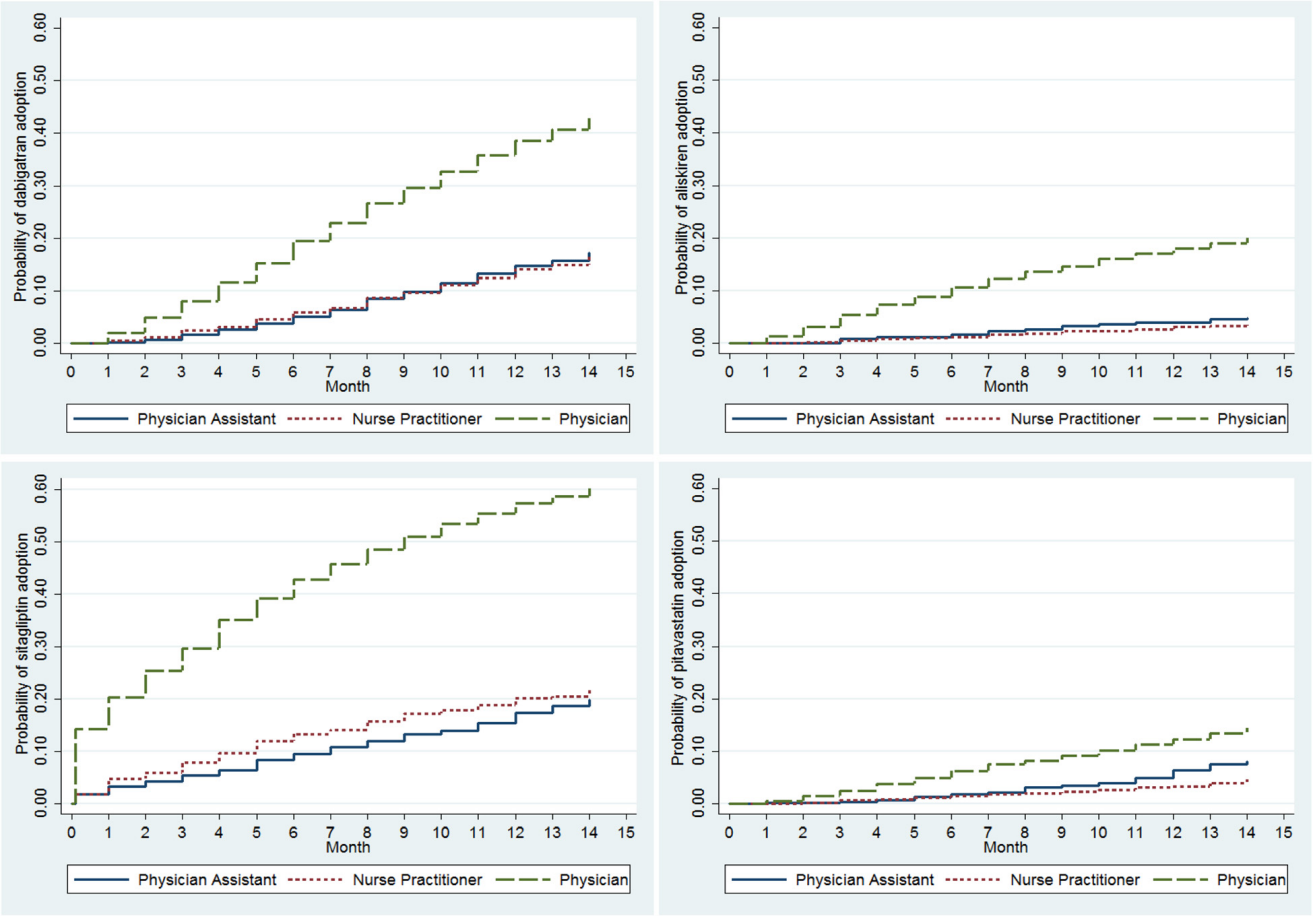
Time to adoption of new chronic disease medications post-FDA approval among primary care providers by specialty^a^. ^a^ *P* < 0.001 log-rank test for all curves

By the final year of the study period (2011), more PCPs had prescribed each of the newly approved chronic disease medications than NPs and PAs (Fig. 1; Table 3). For example, 44.3 % of PCPs ordered at least one prescription for dabigatran compared to 18.5 % of NPs and 20.0 % of PAs (Fig. 1). We found similar differences for each of the other new medications (31.9 % vs. 13.9 % vs. 20.4 % for aliskiren among PCPs, NPs, and PAs; 87.3 % vs. 71.5 % vs. 72.5 % for sitagliptin or saxagliptin among PCPs, NPs, and PAs; and 17.1 % vs. 6.9 % vs. 12.0 % for pitavastatin among PCPs, NPs, and PAs). Across all medication classes, the new cardiovascular agents accounted for a larger share of all prescriptions in the class for PCPs followed by PAs, followed by NPs (Table 3). For example, dabigatran prescriptions accounted for 4.9 % of all anticoagulant prescriptions ordered by PCPs compared to 3.2 % for PAs and 2.8 % for NPs. Similarly, PCPs adopted each of the newly approved cardiovascular medications significantly more rapidly than did NPs and PAs during the post-FDA approval period (Fig. 2; *P* < 0.001). Mean time-to-adoption for the newly approved medications was shorter for PCPs compared to NPs and PAs: dabigatran, 7.3 vs. 8.2 vs. 8.5 months; aliskiren, 6.6 vs. 9.0 vs. 7.9 months; sitagliptin, 4.4 vs. 5.7 vs. 6.9; saxagliptin, 8.4 vs. 10.1 vs. 9.7; and pitavastatin, 7.6 vs. 8.8 vs. 9.5.

**Table 3:** Proportion of prescriptions in each category accounted for by the newly approved medications, 2011

Primary care provider type^a^	Total number of prescriptions, *N* (% of all prescriptions in medication class)
	Dabigatran
Nurse practitioner (*N* = 383)	280 (2.8)
Physician assistant (*N* = 451)	374 (3.2)
Primary care physician (*N* = 4148)	13,261 (4.9)
	Aliskiren
Nurse practitioner (*N* = 469)	478 (0.5)
Physician assistant (*N* = 548)	707 (0.6)
Primary care physician (*N* = 4338)	16,793 (0.8)
	Sitagliptin/saxagliptin
Nurse practitioner (*N* = 435)	6126 (11.7)
Physician assistant (*N* = 527)	7895 (12.7)
Primary care physician (*N* = 4312)	153,926 (13.0)
	Pitavastatin
Nurse practitioner (*N* = 451)	145 (0.2)
Physician assistant (*N* = 526)	299 (0.4)
Primary care physician (*N* = 4299)	6367 (0.5)

^a^N indicates the total number of each provider type regularly prescribing each medication class

#### Sensitivity analyses: only female providers

When sub-setting the analyses to only female providers, the results were largely unchanged – female PCPs adopted the new drugs more rapidly than did NPs and PAs (Additional file 1: Tables S4-S6, Figures S1 and S2). However, there were some noteworthy differences between the main analysis and the sensitivity analysis. Across the included medication classes, the proportion of prescriptions written by female NPs and PAs was greater than in the main analysis (Additional file 1: Table S5). In addition, consistent with the main analysis, by the final year of the study period (2011), dabigatran accounted for a larger share of all prescriptions in the class for female PCPs followed by female PAs, followed by female NPs (Additional file 1: Table S6). In contrast to the main analysis, this pattern did not follow for aliskiren, sitagliptin/saxagliptin, or pitavastatin, for which female PAs accounted for the largest share of prescriptions of the new medications. By the final year of the study period (2011), more female PCPs had prescribed each of the newly approved chronic disease medications than female NPs and PAs, with the exception of pitavastatin (Additional file 1: Figure S1). Similarly, female PCPs adopted each of the newly approved cardiovascular medications significantly more rapidly than did female NPs and PAs during the post-FDA approval period (Additional file 1: Figure S2; *P* < 0.01).

## Discussion

Our exploratory study yielded three key findings regarding NP, PA, and PCP prescribing of newly approved chronic disease medications in the primary care setting. First, we found that NPs and PAs ordered a small but increasing share of prescriptions in some of the most widely used classes, responsible for almost 1 in 10 prescriptions in these classes by 2011. Second, we found that PCPs adopted each of the new chronic disease medications more rapidly than did NPs and PAs, regardless of drug novelty. Third, we found that PCPs ordered a greater proportion of their prescriptions for the new medications than did NPs and PAs. Differences in the rate and speed of drug adoption between PCPs and NPs and PAs may have important implications for care and overall costs at the population level as NPs and PAs continue to take on a larger role in prescribing.

To our knowledge, this study is the first to examine new drug adoption across primary care provider types. This is noteworthy because much of the existing literature comparing NPs and PAs and PCPs has focused on process measures (e.g., patient satisfaction), with less emphasis on utilization measures. For example, a systematic review comparing NPs to PCPs found that processes of care were not significantly different between the two provider types for chronic disease management [21]. Moreover, a Cochrane Review evaluating the impact of doctor-nurse substitution in primary care on patient outcomes, processes of care, and resource utilization reported that appropriately trained NPs can produce as high quality care as PCPs on the measured processes and achieve comparable health outcomes [7]. However, the review acknowledged major limitations in the current literature, including only one study being powered to assess equivalence of care, several studies having methodological limitations, and patient follow-up being short. [7] The most comparable study to ours (not included in the Cochrane Review) examined use of diagnostic imaging ordered by NPs and PAs relative to that of PCPs following office-based encounters as a measure of healthcare utilization. Hughes et al. found that NPs and PAs ordered more imaging services than PCPs for similar patients and concluded that although these differences appeared modest at the individual level, the potential implications on care and costs at the population level are significant [5]. Prior research from the National Ambulatory Medical Care Surveys found that NPs and PAs and PCPs prescribe a similar number of medications per visit [4]. However, our study revealed differential prescribing behavior between NPs and PAs and PCPs for newly approved drugs.

The differences in prescribing we identify may be due to patient-, practice-, provider-, manufacturer-, or payer-level factors that differ between provider types. Previous research has shown that NPs and PAs generally spend greater amounts of time educating and counseling patients and that physicians retain older, more complex patients [22]. Our findings are consistent in that PCPs were more likely than NPs and PAs to prescribe to older patients, who often take multiple medications due to chronic co-morbidity. Given an older, more complex patient panel, PCPs may be more likely to prescribe from a broader prescription armamentarium, including newly approved drugs. In addition, some evidence points to provider sex as an important factor in health technology adoption; male providers tend to adopt new treatment more rapidly on average [19]. Our findings support this notion, given that the vast majority of NPs and PAs were female compared to one-third of PCPs. To explore this, we conducted post-hoc sensitivity analyses by including only female NPs, PAs, and PCPs in order to qualitatively compare these results with the main results. These results were largely similar to the main results, with a few exceptions. On the whole, we continued to find statistically significant differences in the rate of new drug adoption across provider type. Further, differences in peer influences, which may affect new technology adoption, could differ between NPs, PAs, and PCPs [23–25]. It is interesting to note, however, that NPs and PAs in our study worked in much larger practices where there are presumably more peers to learn from and, yet, they were found to be slower adopters of new drugs. Another factor that could drive new drug adoption is pharmaceutical marketing. However, previous research has shown that NPs have similar exposure to pharmaceutical promotion and tend to respond in the same way as physicians [4]. We also note that there were differences in speed and extent of adoption across the five study drugs, with first-in-class drugs being more rapidly adopted than others. Finally, we found that NPs and PAs were more likely to prescribe to patients receiving Medicaid coverage, and it is possible that Medicaid formularies were more restrictive, thus explaining a lower use of new brand name drugs.

Our study has important limitations. First, we lacked clinical information on patient-level factors (e.g., comorbidities). Prescriber adoption decisions may be influenced by their patient case mix although prior studies suggest this explains little of the variation in choice of prescription drugs [26]. Second, we lacked data on the provider characteristics, such as age, training, and years in practice. As previously mentioned, data exist for physician characteristics (e.g., age, sex, medical school, and residency program) in the American Medical Association Physician Masterfile; however, an equivalent dataset does not exist for NPs and PAs. As a result, we were unable to measure these provider-level characteristics. Third, because of a lack of data, we were unable to adjust for some of the external influences on prescribing behavior, such as manufacturer promotional efforts directed at providers, characteristics of the specific organizations in which providers practice, and health plan coverage of other medications. Fourth, our analysis included five newly approved medications from four medication classes and, thus, may not represent prescribing habits of primary care providers for all newly approved medications. Finally, we were not able to assess appropriateness for the prescribing behaviors and thus cannot say whether there is underuse of new drugs by NPs and PAs and/or overuse by PCPs.

## Conclusions

In conclusion, our exploratory findings point to differential adoption of newly approved chronic disease medications between NPs and PAs and PCPs. PCPs were more likely to prescribe each of the newly approved medications, including both more and less novel drugs, by all three measures of adoption used in our study. While there were small absolute differences among prescriber groups detected, these may have important implications for care and overall costs at the population level given the number of new drugs approved each year.

## Abbreviations

HCOS, Healthcare Organization Services; NP, nurse practitioner; PA, physician assistant; PCP, primary care physician

## Additional file

Additional file 1: Table S1. Characteristics of nurse practitioners, physician assistants, and primary care physicians who prescribed anticoagulant prescription medications in Pennsylvania, 2011. **Table S2.** Characteristics of nurse practitioners, physician assistants, and primary care physicians who prescribed oral hypoglycemic prescription medications in Pennsylvania, 2011. **Table S3.** Characteristics of nurse practitioners, physician assistants, and primary care physicians who prescribed HMG-CoA reductase inhibitor prescriptions in Pennsylvania, 2011. **Table S4.** Characteristics of female nurse practitioners, physician assistants, and primary care physicians who prescribed select antihypertensive prescription medications in Pennsylvania, 2011. **Table S5.** Proportion of cardiovascular prescriptions ordered by female nurse practitioners, physician assistants, and primary care physicians over time, 2007–2011. **Table S6.** Proportion of prescriptions from female providers in each category accounted for by the newly approved medications, 2011. **Figure S1.** Percent of each female provider type with any prescription for a newly approved medication in 2011. **Figure S2.** Time to adoption of new chronic disease medications post-FDA approval among female primary care providers. (DOCX 168 kb)
