# Supplementary material for: New chronic disease medication prescribing by nurse practitioners, physician assistants, and primary care physicians: a cohort study
Source: BMC Health Serv Res. 2016 Jul 27;16:312. doi: 10.1186/s12913-016-1569-1 (PMC4964005; doi:10.1186/s12913-016-1569-1)
Supplement: Additional file 1: Table S1. — Characteristics of nurse practitioners, physician assistants, and primary care physicians who prescribed anticoagulant prescription medications in Pennsylvania, 2011. Table S2. Characteristics of nurse practitioners, physician assistants, and primary care physicians who prescribed oral hypoglycemic prescription medications in Pennsylvania, 2011. Table S3. Characteristics of nurse practitioners, physician assistants, and primary care physicians who prescribed HMG-CoA reductase inhibitor prescriptions in Pennsylvania, 2011. Table S4. Characteristics of female nurse practitioners, physician assistants, and primary care physicians who prescribed select antihypertensive prescription medications in Pennsylvania, 2011. Table S5. Proportion of cardiovascular prescriptions ordered by female nurse practitioners, physician assistants, and primary care physicians over time, 2007–2011. Table S6. Proportion of prescriptions from female providers in each category accounted for by the newly approved medications, 2011. Figure S1. Percent of each female provider type with any prescription for a newly approved medication in 2011. Figure S2. Time to adoption of new chronic disease medications post-FDA approval among female primary care providers. (DOCX 168 kb) [file 12913_2016_1569_MOESM1_ESM.docx]

**Additional file 1**

**Table S1. Characteristics of nurse practitioners, physician assistants, and primary care physicians who prescribed anticoagulant prescription medications in Pennsylvania, 2011^*^**

| **Characteristics** | **Nurse Practitioner** | **Physician Assistant** | **Primary Care Physician** |
| --- | --- | --- | --- |
|  | N=441 | N=529 | N=4329 |
| Provider sex, % | -- | -- | -- |
| Female | 93.2 | 76.9 | 33.9 |
| Provider setting, % | -- | -- | -- |
| Rural | 20.4 | 28.4 | 12.0 |
| Provider prescribing | -- | -- | -- |
| Annual prescription volume, mean (sd) | 70.1 | 66.0 | 132.3 |
| Patient age, % | -- | -- | -- |
| <64 | 38.8 | 40.3 | 34.6 |
| 65-74 | 21.0 | 22.6 | 21.7 |
| 75-84 | 24.7 | 23.6 | 25.9 |
| 85+ | 15.5 | 13.5 | 17.8 |
| Source of payment for prescription, % | -- | -- | -- |
| Cash | 4.0 | 4.9 | 5.4 |
| Commercial | 48.1 | 46.5 | 49.3 |
| Medicaid | 6.5 | 7.1 | 4.7 |
| Medicare | 41.4 | 41.5 | 40.6 |
| Primary care medical group | -- | -- | -- |
| Number of practice sites | 487 | 521 | 3268 |
| Number of providers per site, mean (sd) | 49.5 (171.1) | 45.5 (143.9) | 20.7 (89.1) |

^*^*P* <0.001 for comparisons across providers for all variables**Table S2. Characteristics of nurse practitioners, physician assistants, and primary care physicians who prescribed oral hypoglycemic prescription medications in Pennsylvania, 2011^*^**

| **Characteristics** | **Nurse Practitioner** | **Physician Assistant** | **Primary Care Physician** |
| --- | --- | --- | --- |
|  | N=486 | N=582 | N=4442 |
| Provider sex, % | -- | -- | -- |
| Female | 93.6 | 77.2 | 34.0 |
| Provider setting, % | -- | -- | -- |
| Rural | 21.4 | 27.3 | 12.0 |
| Provider prescribing | -- | -- | -- |
| Annual prescription volume, mean (sd) | 343.8 | 409.7 | 635.8 |
| Patient age, % | -- | -- | -- |
| <64 | 59.1 | 59.9 | 47.6 |
| 65-74 | 21.2 | 21.5 | 24.1 |
| 75-84 | 14.1 | 13.0 | 19.2 |
| 85+ | 5.6 | 5.6 | 9.1 |
| Source of payment for prescription, % | -- | -- | -- |
| Cash | 4.1 | 4.3 | 4.3 |
| Commercial | 49.2 | 47.9 | 51.4 |
| Medicaid | 12.4 | 12.9 | 7.4 |
| Medicare | 34.3 | 34.9 | 36.9 |
| Primary care medical group | -- | -- | -- |
| Number of practice sites | 531 | 565 | 3311 |
| Number of providers per site, mean (sd) | 46.3 (163.3) | 42.7 (136.5) | 20.6 (88.5) |

^*^*P* <0.001 for comparisons across providers for all variables **Table S3. Characteristics of nurse practitioners, physician assistants, and primary care physicians who prescribed HMG-CoA reductase inhibitor prescriptions in Pennsylvania, 2011^*^**

| **Characteristics** | **Nurse Practitioner** | **Physician Assistant** | **Primary Care Physician** |
| --- | --- | --- | --- |
|  | N=486 | N=574 | N=4394 |
| Provider sex, % | -- | -- | -- |
| Female | 93.0 | 77.2 | 34.3 |
| Provider setting, % | -- | -- | -- |
| Rural | 20.8 | 27.5 | 11.8 |
| Provider prescribing | -- | -- | -- |
| Annual prescription volume, mean (sd) | 431.7 | 439.6 | 776.4 |
| Patient age, % | -- | -- | -- |
| <64 | 56.3 | 56.1 | 45.4 |
| 65-74 | 21.2 | 21.3 | 22.9 |
| 75-84 | 14.2 | 14.7 | 19.1 |
| 85+ | 6.9 | 6.9 | 10.8 |
| Source of payment for prescription, % | -- | -- | -- |
| Cash | 4.2 | 4.4 | 4.9 |
| Commercial | 52.4 | 50.5 | 52.0 |
| Medicaid | 9.5 | 10.1 | 6.2 |
| Medicare | 33.9 | 35.2 | 36.8 |
| Primary care medical group | -- | -- | -- |
| Number of practice sites | 532 | 554 | 3270 |
| Number of providers per site, mean (sd) | 44.3 (158.9) | 44.1 (140.1) | 20.7 (89.1) |

^*^*P* <0.001 for comparisons across providers for all variables

**Table S4. Characteristics of female nurse practitioners, physician assistants, and primary care physicians who prescribed select antihypertensive^a^ prescription medications in Pennsylvania, 2011^b^**

| **Characteristics** | **Nurse Practitioner** | **Physician Assistant** | **Primary Care Physician** |
| --- | --- | --- | --- |
|  | N=469 | N=454 | N=1511 |
| Provider setting, % | -- | -- | -- |
| Rural | 19.6 | 27.5 | 9.33 |
| Provider prescribing | -- | -- | -- |
| Annual prescription volume, mean (sd) | 417.5 (490.5) | 401.3 (485.3) | 803.3 (654.4) |
| Patient age, % | -- | -- | -- |
| <64 | 59.3 | 60.1 | 50.0 |
| 65-74 | 20.1 | 20.1 | 22.3 |
| 75-84 | 13.1 | 12.9 | 17.4 |
| 85+ | 7.5 | 6.9 | 10.3 |
| Source of payment for prescription, % | -- | -- | -- |
| Cash | 5.4 | 5.3 | 5.0 |
| Commercial | 55.7 | 55.3 | 54.5 |
| Medicaid | 8.5 | 9.1 | 6.7 |
| Medicare | 30.4 | 30.2 | 33.8 |
| Primary care medical group | -- | -- | -- |
| Number of practice sites | 512 | 478 | 1545 |
| Number of providers per site, mean (sd) | 45.2 (163.5) | 53.0 (158.2) | 39.0 (125.5) |

^a^Antihypertensive medications included those targeting the renin-angiotensin-aldosterone system, including angiotensin-converting enzyme inhibitors, angiotensin II receptor blockers, and direct renin inhibitors; ^b^*P* <0.001 for comparisons across providers for all variables

**Table S5. Proportion of cardiovascular prescriptions ordered by female nurse practitioners, physician assistants, and primary care physicians over time, 2007—2011**

|  | **2007** | **2008** | **2009** | **2010** | **2011** |
| --- | --- | --- | --- | --- | --- |
| Antihypertensives | | | | | |
| Total prescriptions, N | 535,942 | 575,346 | 613,199 | 666,368 | 705,578 |
| NP (%) | 7.1 | 8.6 | 9.8 | 11.5 | 12.2 |
| PA (%) | 5.9 | 7.2 | 8.5 | 10.1 | 11.5 |
| PCP (%) | 87.0 | 84.2 | 81.7 | 78.4 | 76.3 |
| Anticoagulants | | | | | |
| Total, N | 59,293 | 62,376 | 66,748 | 74,654 | 80,790 |
| NP | 5.9 | 7.3 | 8.3 | 10.5 | 10.7 |
| PA | 4.3 | 6.0 | 7.9 | 9.5 | 10.9 |
| PCP | 89.9 | 86.6 | 83.8 | 80.0 | 78.4 |
| Oral hypoglycemics | | | | | |
| Total, N | 320,801 | 336,021 | 357,585 | 379,088 | 390,593 |
| NP | 7.0 | 8.5 | 9.8 | 11.7 | 12.2 |
| PA | 5.8 | 6.9 | 8.4 | 10.0 | 11.6 |
| PCP | 87.2 | 84.6 | 81.8 | 78.3 | 76.2 |
| HMG-CoA Reductase inhibitors | | | | | |
| Total, N | 338,368 | 361,447 | 396,540 | 435,148 | 468,689 |
| NP | 7.4 | 9.0 | 10.4 | 12.0 | 12.5 |
| PA | 6.0 | 7.0 | 8.4 | 10.2 | 11.8 |
| PCP | 86.6 | 84.0 | 81.2 | 77.8 | 75.8 |

Abbreviations: NP, nurse practitioner; PA, physician assistant; PCP, primary care physician

**Table S6. Proportion of prescriptions from female providers in each category accounted for by the newly approved medications, 2011**

| Primary care provider type^a^ | Total number of prescriptions, N  (% of all prescriptions in medication class) |
| --- | --- |
|  | Dabigatran |
| Nurse practitioner (N=356) | 237 (2.7) |
| Physician assistant (N=347) | 278 (3.2) |
| Primary care physician (N=1,388) | 2,542 (4.0) |
|  | Aliskiren |
| Nurse practitioner (N=436) | 433 (0.5) |
| Physician assistant (N=419) | 574 (0.7) |
| Primary care physician (N=1,474) | 2,912 (0.5) |
|  | Sitagliptin/saxagliptin |
| Nurse practitioner (N=406) | 5,586 (11.7) |
| Physician assistant (N=409) | 6,026 (13.3) |
| Primary care physician (N=1,451) | 36,876 (12.4) |
|  | Pitavastatin |
| Nurse practitioner (N=421) | 123 (0.2) |
| Physician assistant (N=412) | 250 (0.5) |
| Primary care physician (N=1,454) | 1,175 (0.3) |

^a^N indicates the total number of each provider type regularly prescribing each medication class

**Figure S1. Percent of each female provider type with any prescription for a newly approved medication in 2011^a^**


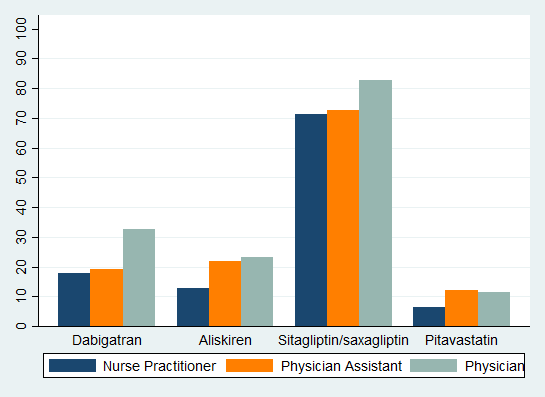


^a^Prevalence of any prescription of newly approved chronic disease medications among primary care providers prescribing any drug from the medication class

**Figure S2. Time to adoption of new chronic disease medications post-FDA approval among female primary care providers by specialty^a^**


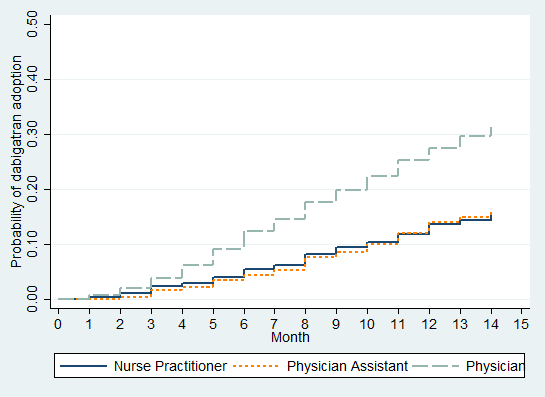

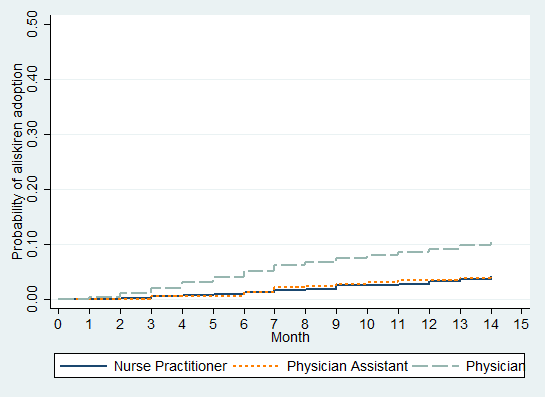

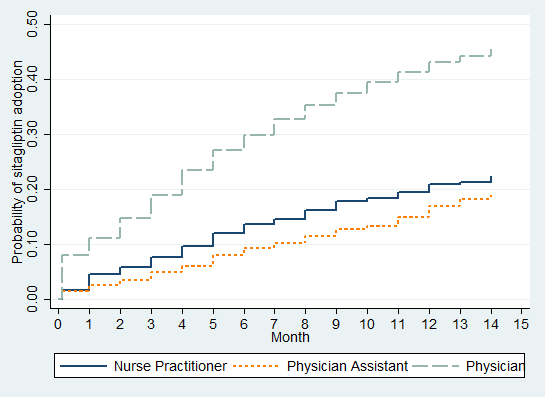

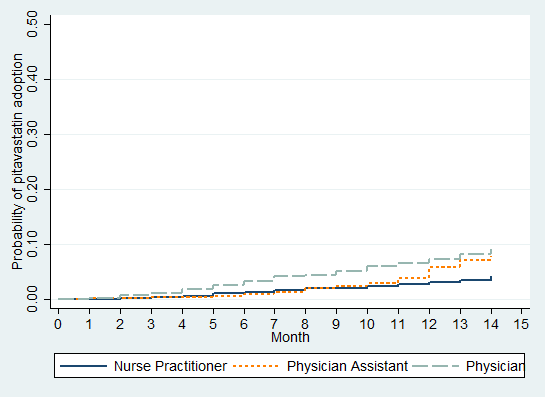


^a^*P*<0.01 log-rank test for all curves
